# Lateral versus conventional fasciotomy for prevention of lateral femoral cutaneous nerve injury in total hip arthroplasty with direct anterior approach: a study protocol for a dual-center, double-blind, randomized controlled trial

**DOI:** 10.1186/s13063-022-06496-2

**Published:** 2022-07-15

**Authors:** Hiroki Tanabe, Tomonori Baba, Yu Ozaki, Naotake Yanagisawa, Sammy Banno, Taiji Watari, Yasuhiro Homma, Masashi Nagao, Kazuo Kaneko, Muneaki Ishijima

**Affiliations:** 1 Department of Orthopedic Surgery, Juntendo University, 2-1-1 Hongo, Bunkyo-ku, Tokyo, Japan; 2 Department of Orthopedic Surgery, Juntendo Tokyo Koto Geriatric Medical Center, 3-3-20 Shinsuna, Koutouku, Tokyo, Japan; 3 Medical Technology Innovation Center, Juntendo University, 2-1-1 Hongo, Bunkyo-ku, Tokyo, Japan

**Keywords:** Total hip arthroplasty, Direct anterior approach, Lateral femoral cutaneous nerve, Ultrasonography

## Abstract

**Background:**

An incision for total hip arthroplasty (THA) via the direct anterior approach (DAA) is generally made outside of the space between the sartorius and tensor fasciae latae muscles to prevent lateral femoral cutaneous nerve (LFCN) injury. Anatomical studies have revealed that the LFCN courses between the sartorius and tensor fasciae latae muscles. When the LFCN branches radially while distributing in the transverse direction from the sartorius muscle to the tensor fasciae latae muscle, it is called the fan type. Studies suggest that damage to the fan type LFCN is unavoidable during conventional fasciotomy.

We previously demonstrated that injury to non-fan variation LFCN occurred in 28.6% of patients who underwent THA by fasciotomy performed 2 cm away from the intermuscular space. This suggests that the conventional approach also poses a risk of LFCN injury to non-fan variation LFCN. LFCN injury is rarely reported in the anterolateral approach, which involves incision of fascia further away than the DAA. The purpose of this study is to investigate how the position of fasciotomy in DAA affects the risk of LFCN injury.

**Methods:**

We will conduct a prospective, randomized, controlled study. All patients will be divided into a fan variation and a non-fan variation group using ultrasonography before surgery. Patients with non-fan variation LFCN will receive conventional fasciotomy and lateral fasciotomy in the order specified in the allocation table created in advance by our clinical trial center. The primary endpoint will be the presence of LFCN injury during an outpatient visit using a patient-based questionnaire. The secondary endpoints will be assessed based on patient-reported outcomes at 3 months after surgery in an outpatient setting using the Western Ontario and McMaster Universities Osteoarthritis Index, the Japanese Orthopaedic Association Hip-disease Evaluation Questionnaire, and the Forgotten-Joint Score-12.

**Discussion:**

We hypothesize that the incidence of LFCN injury due to DAA-THA is reduced by making the incision further away from where it is typically made in conventional fasciotomy.

If our hypothesis is confirmed, it will reduce the disadvantages of DAA and improve patient satisfaction.

**Trial registration:**

UMIN Clinical Trials Registry, UMIN000035945. Registered on 20 February, 2019.

## Background

Total hip arthroplasty (THA) is an effective surgical procedure that helps reduce pain and improve function of patients with hip disorders [1, 2]. There are several surgical approaches for THA; in particular, direct anterior approach (DAA), an intermuscular and internervous approach, causes less harm to the soft tissue compared with other approaches. Thus, DAA is associated with a lower risk of dislocation and rapid recovery of muscular strength after the procedure [3, 4]. However, it may result in injury to the lateral femoral cutaneous nerve (LFCN) [5–9]. The LFCN is a pure sensory nerve that controls the anterolateral aspect of the thighs, and LFCN injury leads to hypesthesia and dysesthesia. Several studies demonstrated that LFCN injury does not affect hip joint function following THA [6, 10, 11]; however, even in such cases, sensory disturbance caused by LFCN injury may affect patient satisfaction.

The LFCN passes posterolaterally from the iliopsoas and under the inguinal ligament before it crosses between the sartorius and tensor fasciae latae muscles [12, 13]. Thus, in order to prevent LFCN injury, an incision for DAA is generally made outside of the space between the sartorius and tensor fasciae latae muscles. However, LFCN injury has been reported in 0.1 to 81% of patients [1, 6, 12, 14]. In addition to the LFCN that passes in between these muscles, Rudin et al. performed pathological examinations and identified a different type of the LFCN that radially distributes in the transverse direction from the sartorius muscle into the tensor fasciae latae muscles [15]. This variation of the LFCN, which passes across the muscles, is called the fan type, and studies suggest that damage to the fan type LFCN is unavoidable by conventional fasciotomy [15]. Moreover, we previously demonstrated that injury to non-fan type LFCN (LFCN that passes in between the sartorius and tensor fasciae latae muscles) occurred in 28.6% of patients who underwent THA by fasciotomy performed 2 cm away from the intermuscular space [16]. This suggests that the conventional approach also poses a risk of LFCN injury for non-fan variation LFCN.

In the anterolateral approach (ALA), the incision is made further away from the fascia in order to reach the hip joint from between the tensor fasciae latae and gluteus medius. As with DAA, ALA is also an inter-muscular approach; thus, it enables preservation of the soft tissue and is associated with a relatively low risk of dislocation and rapid recovery of muscular strength after the procedure [17–21]. However, as both the tensor fasciae latae and gluteus medius are supplied by a motor nerve called the superior gluteal nerve, damage to the superior gluteal nerve due to ALA may reduce muscular strength [22, 23]. On the other hand, unlike DAA, LFCN injury is rarely reported after ALA [23].

Based on these findings, we hypothesized that the incidence of LFCN injury due to DAA-THA may be reduced by making the incision further away from where it is typically made in conventional fasciotomy. In this prospective study, we investigated how the position of fasciotomy in DAA-THA affects the risk of LFCN injury.

## Methods

### Study setting

This is a dual-center, double-blind, prospective randomized controlled two-arm trial with parallel group design in a 1:1 allocation ratio. The study is conducted in the Juntendo University Hospital and the Juntendo Tokyo Koto Geriatric Medical Center in Japan.

### Ethics and informed consent

The study was approved by the Research Ethics Board of Juntendo University Hospital in January 2019 and was subsequently registered in the University Hospital Medical Information Network (UMIN) clinical trials registry on February 20, 2019 (ID: UMIN000035945). The study team will explain about the study in writing and verbally, and voluntary written consent will be obtained from the patients. Patients will be informed immediately if any new information that may affect consent is obtained or if any changes to the study protocol that may affect consent will be implemented and will have the opportunity to decide whether they wish to continue participating in the study. We will also revise the consent form, and upon approval from the hospital’s research ethics board, we will provide the revised form to the study subjects to receive consent.

### Eligibility criteria

Patients aged 20 to 90 years who will undergo primary THA using the DAA, have a non-fan variation LFCN confirmed by ultrasonography before THA, and will have provided written informed consent for participation will be eligible. Patients with hip trauma or infection, body mass index ≥ 35, a history of hip surgery, inability to follow physician instructions, and those deemed unsuitable for inclusion by the principal investigator, such as neurological disorder, illiterate patients, or patients with severe medical conditions, will not be enrolled.

### Sample size

A sample size of 144 subjects is planned. According to our previous study, the overall incidence of LFCN injury incidence after DAA for THA is 42%, and the incidence of non-fan variation is 28.6% [16]. Thus, we hypothesize the lateral DAA approach will reduce the rate of LFCN injury to 10%. Using an alpha error of 5% and a power of 80% in two-tailed analysis, 124 subjects need to be recruited. These calculations were performed using SAS software version 9.4 (SAS Institute, Cary, NC, USA). Considering the dropout, we will enroll a minimum of 72 patients in each group.

### Recruitment and allocation

**Fig. 1:**
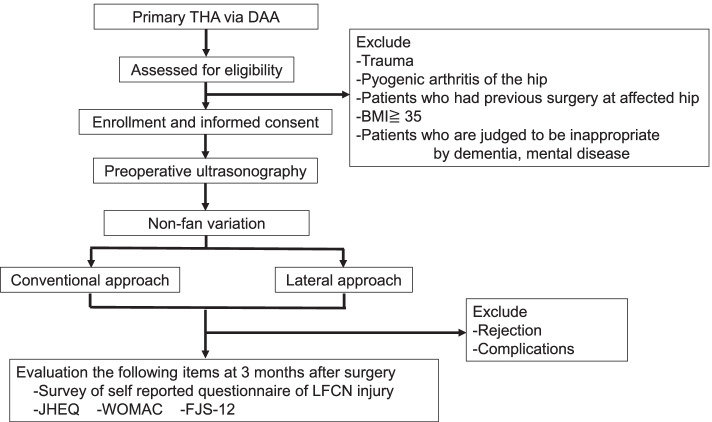
Flow chart of the trial protocol

Patients who meet the eligibility criteria and have a non-fan variation LFCN confirmed by ultrasonography will be enrolled after written informed consent is obtained. Participants will be enrolled by the surgeon and assigned to interventions by the ultrasound inspector. Ultrasonography will be performed in the operating room before surgery to classify the LFCN as fan variation or non-fan variation. The surgeon will confirm the adherence of ultrasound protocol. After ultrasonography, the inspector will direct the surgeon to perform a lateral or conventional DAA according to the allocation table. The allocation table will be created in advance by a statistician and use a permuted block randomization schedule with a fixed block size of 4. The table will be stored by the ultrasound inspectors and concealed until the patient is assigned. Surgery will be performed as directed by the inspector. The trial flow chart is shown in Fig. 1.

### Blinding

The statistician, participants, follow-up observer, and data analyst will be blinded to the surgical approach used in each participant. However, surgeons cannot be blinded because of their participation in the procedure.

### Study timeline and data collection

**Fig. 2:**
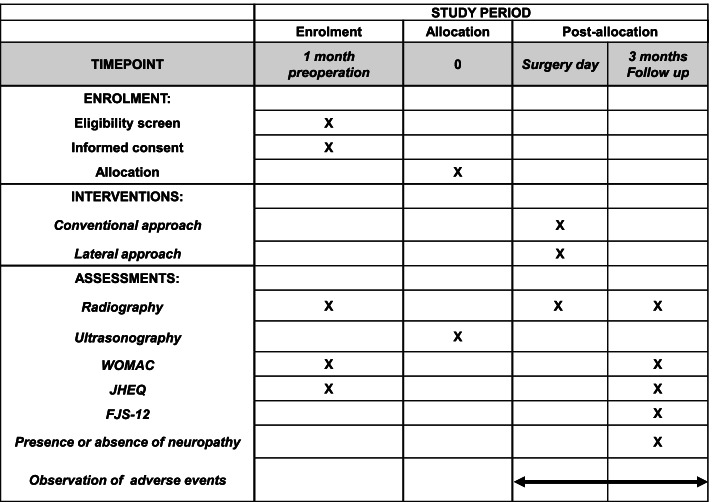
The schedule of enrolment, intervention, and assessments. WOMAC, Western Ontario and McMaster Universities Osteoarthritis Index; JHEQ, Japanese Orthopaedic Association Hip-disease Evaluation Questionnaire; FJS-12, Forgotten-Joint Score-12

After providing consent, study subjects will participate in the study for a total of 4 months, consisting of a 1-month pre-observation period and a 3-month post-observation period. The timeline for the assessment and data collection regarding primary and secondary outcomes is shown in Fig. 2.

### Ultrasonography of LFCN

**Fig. 3:**
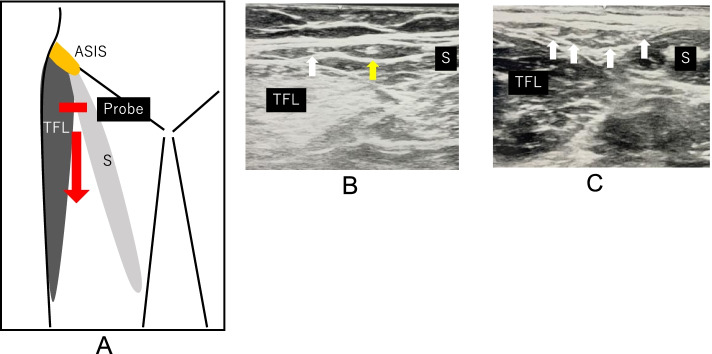
**A** Schematic diagram showing the initial location of the probe. The probe is moved from the proximal to distal hip joint along the region between the sartorius and tensor fasciae latae muscles (arrow). ASIS, anterior superior iliac spine; TFL, tensor fasciae latae; S, sartorius. **B** Transverse ultrasound image of the non-fan variation within the intermuscular space between the sartorius and the tensor fasciae latae (yellow arrow: main branch, White arrow: thin branch). TFL, tensor fasciae latae; S, sartorius. **C** Transverse ultrasound image of the fan variation within the intermuscular space between the sartorius and the tensor fasciae latae (arrows: several nerve branches). TFL, tensor fasciae latae; S, sartorius

Ultrasonography will be performed in the supine position using LOGIQ e Expert (GE health-care, Japan) with a 12-MHz linear array transducer according to the protocol developed by Zhu et al. [24]. First, the transducer will be placed perpendicular to the body axis and 1–2 cm away from the outer edge of the inguinal ligament. At this location, images of the sartorius and tensor fasciae latae muscles will be collected, and the LFCN between the muscles will be identified. While keeping the LFCN in the imaging field, the probe will be moved distally between the sartorius and tensor fasciae latae muscles (Fig. 3A). The LFCN is a structure in between the sartorius and tensor fasciae latae muscles that is characterized by a low signal intensity (nerve bundle) area surrounded by an oval or spindle-shaped high signal intensity (perineurium) area [24]. Fan variation and non-fan variation LFCN will be defined based on the pathological findings reported by Rudin et al. [15]. Specifically, non-fan variation LFCN is defined as a structure with one main nerve surrounded by multiple smaller branches (Fig. 3B) and fan variation LFCN as a structure with three or more branches of similar size or two or more nerves branching out from one main nerve (Fig. 3C) [15].

### Surgical procedures of THA

In all cases, THA will be performed under general anesthesia by one of five experienced (> 50 cases) hip surgeons [9, 25]. The surgery will be performed with at least two research team members, including the surgeon and research coordinators, to ensure that there is adherence to the protocol. Study subjects will be placed in the supine position, and the procedures will be performed with traction beds and intraoperative fluoroscopy [26]. The skin incision will be made 2 cm distally from the superior anterior iliac spine and in parallel with the line that connects the superior anterior iliac spine with the head of the fibula. The total length of the incision will be 10 cm. The same techniques will be used to set up the surgical field and place the implants for both groups. The same implant will be used for both groups.

### Interventions

**Fig. 4:**
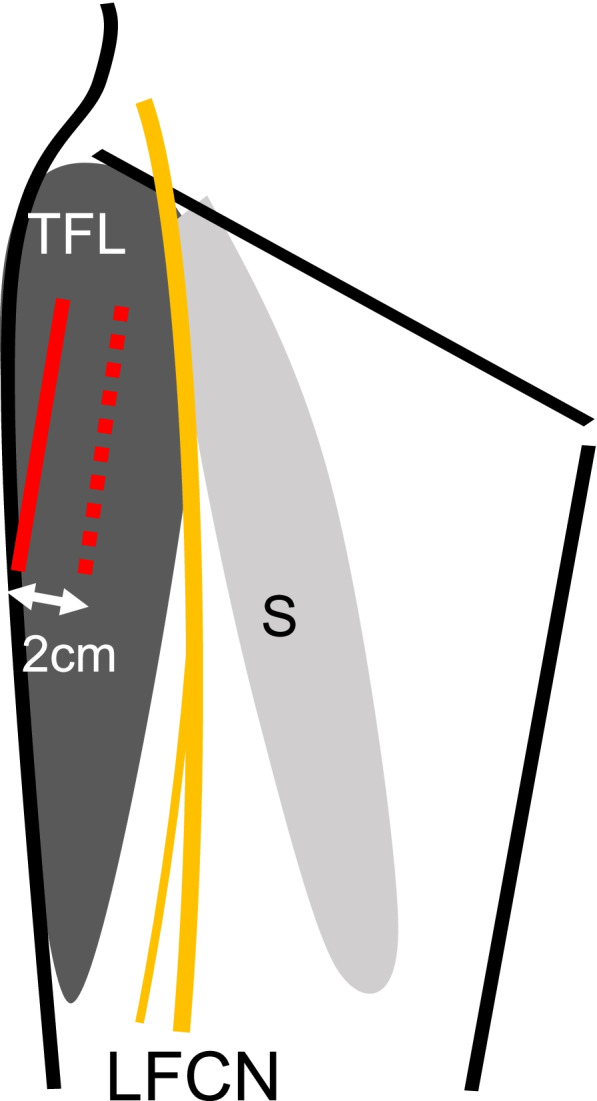
Schematic diagram showing the line of fascia incision in conventional and lateral approach (red solid line: the site of fascia incision in lateral approach, red dotted line: the site of fascia incision in conventional approach). TFL, tensor fasciae latae; S, sartorius; LFCN, lateral femoral cutaneous nerve

The conventional approach involves incision of the skin, followed by incision of the fascia just below the site of skin incision. On the other hand, the lateral approach proceeds outside from the site of skin incision and involves incision of the fascia at a site 2 cm lateral from the conventional site of incision. Incision of the fascia will be performed to the extent that both approaches can be viewed in the surgical field (Fig. 4).

### Standard of care

All patients will receive the standard routine post-operative management protocol of a wound review and a further review using X-ray at postoperative and 3 months follow-up. Postoperative rehabilitation and physiotherapy will be standardized according to our institutional protocol and initiated for all patients at day 1 post-operatively.

### Withdrawal criteria

Study subjects will be withdrawn from the study if the principal investigator and the study team decide that it is impossible to continue the study activities under the study’s criteria. If this applies, the subject will be given an explanation for their withdrawal. The withdrawal criteria will be:

- If withdrawal from the study is requested or if consent is withdrawn by a study subject,
- If the study is terminated, or
- If the principal investigator decides that the study should be terminated for reasons other than those listed above.

Withdrawal patients will continue to receive routine as required. Participants who withdrawal or are withdrawn from the study interventions will be followed up as per intention to treat, unless consent has been fully withdrawn.

### Outcomes and measurements

#### Primary outcomes

The primary outcome of the study will be LFCN injury. Nerve injury will be assessed 3 months after the surgery during an outpatient visit using a patient-based questionnaire. Based on previous studies, we defined LFCN injury as having (1) no sensation, (2) numbness, (3) tingling, or (4) pain in the lateral aspect of the thigh except at the site of the wound [12].

#### Secondary outcomes

The secondary outcome will be assessed based on patient-reported outcomes (PROs) at 3 months after surgery in an outpatient setting to examine the extent to which LFCN injury has affected the patient’s quality of life (QOL) and complications (dislocations, infections, or intraoperative and periprosthetic fracture).

PROs will be evaluated using the Western Ontario and McMaster Universities Osteoarthritis Index (WOMAC) (pain, stiffness, and function subscales), the Japanese Orthopaedic Association Hip-disease Evaluation Questionnaire (JHEQ), and the Forgotten-Joint Score-12 (FJS-12).

The WOMAC was first described by Bellamy and Buchanan in 1986. It uses a scoring system with a total score of 0 (poor) to 96 (excellent) and has since been used worldwide to evaluate hip and knee osteoarthritis. The JHEQ is a self-administered questionnaire for evaluating hip disorders in the Japanese population with a total score of 0 (poor) to 84 (excellent). The FJS-12 is a self-administered questionnaire measuring the PROs on the ability of the patient to forget about their prosthetic implant in everyday life and has a total score of between 0 (poor) and 84 (excellent) [26]. The lowest and highest scores for WOMAC are 0 and 96, whereas those of JHEQ are 0 and 84; thus, the lowest and highest scores for each questionnaire will be converted to 0 and 100.

### Sample size

According to previous studies, the incidence of LFCN injury due to THA with the DAA is 28.6% in non-fan variation patients [16]. Therefore, we hypothesize the lateral DAA approach will reduce the rate of LFCN injury to 15%. Using an alpha of 5% and a power of 80% in two-tailed analysis, 130 subjects need to be recruited. These calculations were performed using SAS software version 9.4 (SAS Institute, Cary, NC, USA). As we expect a 10% dropout rate, we will enroll a minimum of 144 patients in each group.

### Information disclosure

The study was registered in the public UMIN clinical trials registry. The results will be presented at the Japanese Orthopaedic Association Annual Meeting, and we intend to submit them to an orthopedic journal. Data that will be presented will only consist of those that have been processed statistically; thus, personal health information of the study subjects will not be made available.

### Safety and adverse events

We do not anticipate problems that are detrimental to the participants. However, in the event of a serious adverse event (SAE), the principal investigator will take necessary measures, report to the hospital director, and share information with study investigators. Any unexpected SAE that occurs during the course of the study will be reported to the hospital director, study team members, and the Ministry of Health, Labor and Welfare in Japan. Actions taken in response will be announced.

### Declaration of interests

The study will be conducted using internal funding within the Orthopedics and Sports Medicine Departments. The study did not receive any other funding or benefits from external sources; thus, there is no conflict of interest that will affect the analysis or results of the study.

According to the Juntendo University Regulations of Management of Conflicts of Interest, the principal investigator and study team will report any conflict of interest and receive approval by the management committee.

### Statistical analysis

Chi-square test will be used to compare the presence of LFCN injury. We will perform a normality test using Shapiro-Wilk to detect parametric or nonparametric data. To assess the effect of LFCN injury on patient QOL to compare differences in the PRO assessments between lateral and conventional approach, the Mann-Whitney *U* test will be used if data is non-parametric, and the *t*-test will be used if data is parametric.

*P* < 0.05 will be considered significant. We will also present treatment effects and confidence intervals and perform adjusting for confounding factors as an exploratory analysis using logistic regression or multiple regression. Statistical analyses will be performed using SPSS software version 22 for Mac (IBM Corp., Armonk, NY, USA).

### Data protection

In order to protect the confidentiality of personal health information, a study ID that does not contain any identifying information will be assigned to each subject. The principal investigator will follow the Standard Operating Procedures for the Storage of Materials and Information in Medical Research with Human Subjects. All data will be stored electronically or within the Orthopedics Department of our hospital for 5 years from the end (or after termination) of the study. Upon completion of this 5-year period, all documents will be deleted or discarded appropriately.

### Monitoring

Annual monitoring will be performed by the Clinical Research and Trial Center of Juntendo University, Japan, according to the Standard Operating Procedures for Monitoring and Audits in Medical Research with Human Subjects. Monitoring will be independent from conflicting interests.

### Audits

Monitoring and audits will be performed according to the Standard Operating Procedures for Monitoring and Audits in Medical Research with Human Subjects of the Clinical Research Support Center.

The institutional Research Ethics Board will review the study on an annual basis and evaluate whether the study activities can continue. Amendments to the study documents will be submitted each time and approved by the Research Ethics Board. If any severe adverse events are identified within the hospital, the study team will report to the Hospital Director immediately. The Research Ethics Board will then determine whether the study activities need to be terminated.

## Discussion

Surgical approaches for the placement of a hip prosthesis are classified as posterior, lateral, or anterior. DAA and ALA are anterior approaches that do not involve dissection of the tendon to reach the hip joints. Thus, they have advantages over other approaches in terms of functional recovery following the placement of a hip prosthesis [3, 14, 18, 27, 28]. Although both DAA and ALA are intermuscular approaches, ALA is associated with a risk of motor paralysis as it involves approaching between the tensor fasciae latae and gluteus medius, which are supplied by the superior gluteal nerve [22, 23]. On the other hand, the risk of motor paralysis is minimal in DAA as it is the only approach that involves the inter-innervated area where the sartorius and tensor fasciae latae muscles are supplied by the femoral and superior gluteal nerves, respectively [23, 29]. Thus, we selected DAA over ALA for the purpose of this study.

Rudin et al. reported three patterns of LFCN distribution (sartorius-type, posterior-type, and fan type) in an anatomical study and reported that LFCN injury is unavoidable in patients with a fan-type distribution if conventional DAA is used [15]. In our previous study, LFCN injury was observed after surgery in 9 of 10 patients with the fan type based on the ultrasound findings before THA (positive predictive value: 90%); however, no LFCN injury was observed in 25 of 26 patients judged as non-fan type (specificity: 96.2%). These findings suggest that ultrasonography is useful for evaluating LFCN distribution [16]. However, there is no study to detect a fan or non-fan type of LFCN. This will be a limitation.

In order to prevent LFCN, an incision for DAA is generally made outside of the space between the sartorius and tensor fasciae latae muscles. However, 42% of our patients developed LFCN injury [16]. In a subsequent study, we excluded fan type LFCN based on ultrasound findings because injury to fan type LFCN is considered unavoidable. We reported injury to non-fan type LFCN in 28.6% of patients [16]. Based on pathological findings, Ropars et al. suggested that damage to the nerves can be prevented by approaching as laterally and distally as possible [30]. We hypothesize that the risk of injury to non-fan variation LFCN may be reduced by an approach that is similar to ALA whereby the incision is made further away from the location where it is typically performed in conventional fasciotomy. The patients will be blinded by placing the skin incision in the same position in both groups although the position of fascia incision is different. The surgeon cannot be blinded, but the statisticians, participants, follow-up observers, and data analysts will be blinded. If this approach is successful at preventing nerve injury, it will overcome one of the few limitations of DAA. We believe that this will be greatly beneficial to patients undergoing THA.

## Trial status

We were actively recruiting at the time of submission. Patient recruitment started on June 2019 and the study was scheduled for completion in March 2021. The date of enrolment of the first participant to the trial was 3 June, 2019. This protocol is version 1.0, dated 20 February, 2019. The study is scheduled for completion in March 2023.

## Data Availability

The datasets generated and analyzed during this study are available from the corresponding author upon reasonable request. The informed consent form and other related documents are also available from the corresponding author upon request. This trial does not include collection of biological specimens.

## Abbreviations

THA: Total hip arthroplasty
DAA: Direct anterior approach
LFCN: Lateral femoral cutaneous nerve
BMI: Body mass index
QOL: Quality of life
PROs: Patient-reported outcomes
WOMAC: Western Ontario and McMaster Universities Osteoarthritis Index
JHEQ: Japanese Orthopaedic Association Hip-Disease Evaluation Questionnaire
FJS-12: Forgotten-Joint Score-12
